# Untargeted serum metabolomics reveals potential biomarkers and metabolic pathways associated with the progression of gastroesophageal cancer

**DOI:** 10.1186/s12885-023-11744-y

**Published:** 2023-12-15

**Authors:** Jiajing Che, Yongbin Zhao, Bingbing Gu, Shuting Li, Yunfei Li, Keyu Pan, Tiantian Sun, Xinyue Han, Jiali Lv, Shuai Zhang, Bingbing Fan, Chunxia Li, Cheng Wang, Jialin Wang, Tao Zhang

**Affiliations:** 1 Department of Biostatistics, School of Public Health, Cheeloo College of Medicine, Shandong University, Jinan, 250012 Shandong China; 2 Institute for Medical Dataology, Cheeloo College of Medicine, Shandong University, Jinan, 250012 China; 3 Shandong Cancer Hospital and Institute, Shandong First Medical University and Shandong Academy of Medical Sciences, 440 Jiyan Road, Jinan, 250117 China

**Keywords:** Gastroesophageal cancer, Esophageal squamous cell carcinoma, Cancer progression, Metabolomics, Biomarker.

## Abstract

**Background:**

Previous metabolic studies in upper digestive cancer have mostly been limited to cross-sectional study designs, which hinders the ability to effectively predict outcomes in the early stage of cancer. This study aims to identify key metabolites and metabolic pathways associated with the multistage progression of epithelial cancer and to explore their predictive value for gastroesophageal cancer (GEC) formation and for the early screening of esophageal squamous cell carcinoma (ESCC).

**Methods:**

A case-cohort study within the 7-year prospective Esophageal Cancer Screening Cohort of Shandong Province included 77 GEC cases and 77 sub-cohort individuals. Untargeted metabolic analysis was performed in serum samples. Metabolites, with FDR q value < 0.05 and variable importance in projection (VIP) > 1, were selected as differential metabolites to predict GEC formation using Random Forest (RF) models. Subsequently, we evaluated the predictive performance of these differential metabolites for the early screening of ESCC.

**Results:**

We found a distinct metabolic profile alteration in GEC cases compared to the sub-cohort, and identified eight differential metabolites. Pathway analyses showed dysregulation in D-glutamine and D-glutamate metabolism, nitrogen metabolism, primary bile acid biosynthesis, and steroid hormone biosynthesis in GEC patients. A panel of eight differential metabolites showed good predictive performance for GEC formation, with an area under the receiver operating characteristic curve (AUC) of 0.893 (95% CI = 0.816–0.951). Furthermore, four of the GEC pathological progression-related metabolites were validated in the early screening of ESCC, with an AUC of 0.761 (95% CI = 0.716–0.805).

**Conclusions:**

These findings indicated a panel of metabolites might be an alternative approach to predict GEC formation, and therefore have the potential to mitigate the risk of cancer progression at the early stage of GEC.

**Supplementary Information:**

The online version contains supplementary material available at 10.1186/s12885-023-11744-y.

## Introduction

Gastroesophageal cancer (GEC) encompasses tumors in the esophagus, gastroesophageal junction, and stomach [1]. Its insidious presentation and rapid progression typically lead to late-stage diagnosis and treatment, making it a leading cause of digestive cancer mortality [2]. Esophageal squamous cell carcinoma (ESCC), the most common type of esophagus cancer, develops through multiple stages of epithelial cancer, progressing from normal epithelial to low- and high-grade intraepithelial neoplasia, and finally to invasive carcinoma [3]. Esophageal squamous epithelial dysplasia is a recognized precursor to ESCC [4, 5]. Hence, early detection of precancerous lesions is crucial for identifying high-risk groups, enhancing the efficacy of early screening, reducing the risk of GEC, and improving the survival time [6]. While endoscopy with iodine staining is an established technique for identifying esophageal squamous dysplasia, it is still limited by its invasive and costly characteristics [7–9].

Metabolomics has emerged a new platform for biomarker discovery in recent years [10–17]. In our previous study, using an untargeted metabolomics method, good discriminating performances were achieved for the early stage of ESCC, especially in tumor in situ (TIS), with the values of an area under the receiver operating characteristic curve (AUC) 0.939 (95% CI = 0.841-1.000) [13, 14]. Yuan et al. highlighted the significant association of alanine, aspartate, and glutamate (AAG) metabolism with the prevalence and progression of gastric cancer (GC) [18]. However, the majority of these metabolomics studies have been based on cross-sectional or retrospective study designs, either for early screening or cancer diagnosis, which hampered the effective prediction in the early stage of cancer. Prospective studies on pathological progression still need further investigation.

In the present study, we applied untargeted metabolomics to explore serum metabolic profile changes during the GEC formation based on a prospective case-cohort study. We aimed to identify the key metabolites and metabolic pathways associated with the complex multistage epithelial cancer formation in the high-risk area of China. Finally, we explored the predictive value of these serum metabolic biomarkers in GEC formation, and further evaluated their validity in the early screening of ESCC.

## Materials and methods

### Study population

A total of 4558 participants aged 40–69 years were recruited at the Esophageal Cancer Screening Base of Shandong Province (City of Feicheng, Shandong, China) between June 2013 and September 2014 [13]. All participants underwent esophageal cancer screening using endoscopy with mucosal iodine staining. Baseline questionnaires and physical evaluations were conducted to collect sociodemographic information and various health parameters. Serum samples were also collected for metabolomics analysis. After excluding individuals without complete baseline information or those who did not progress beyond mild esophagitis, we established the initial cohort for the case-cohort study (n = 3514; see methods section of the Supplementary Materials and Figure S1).

**Fig. 1:**
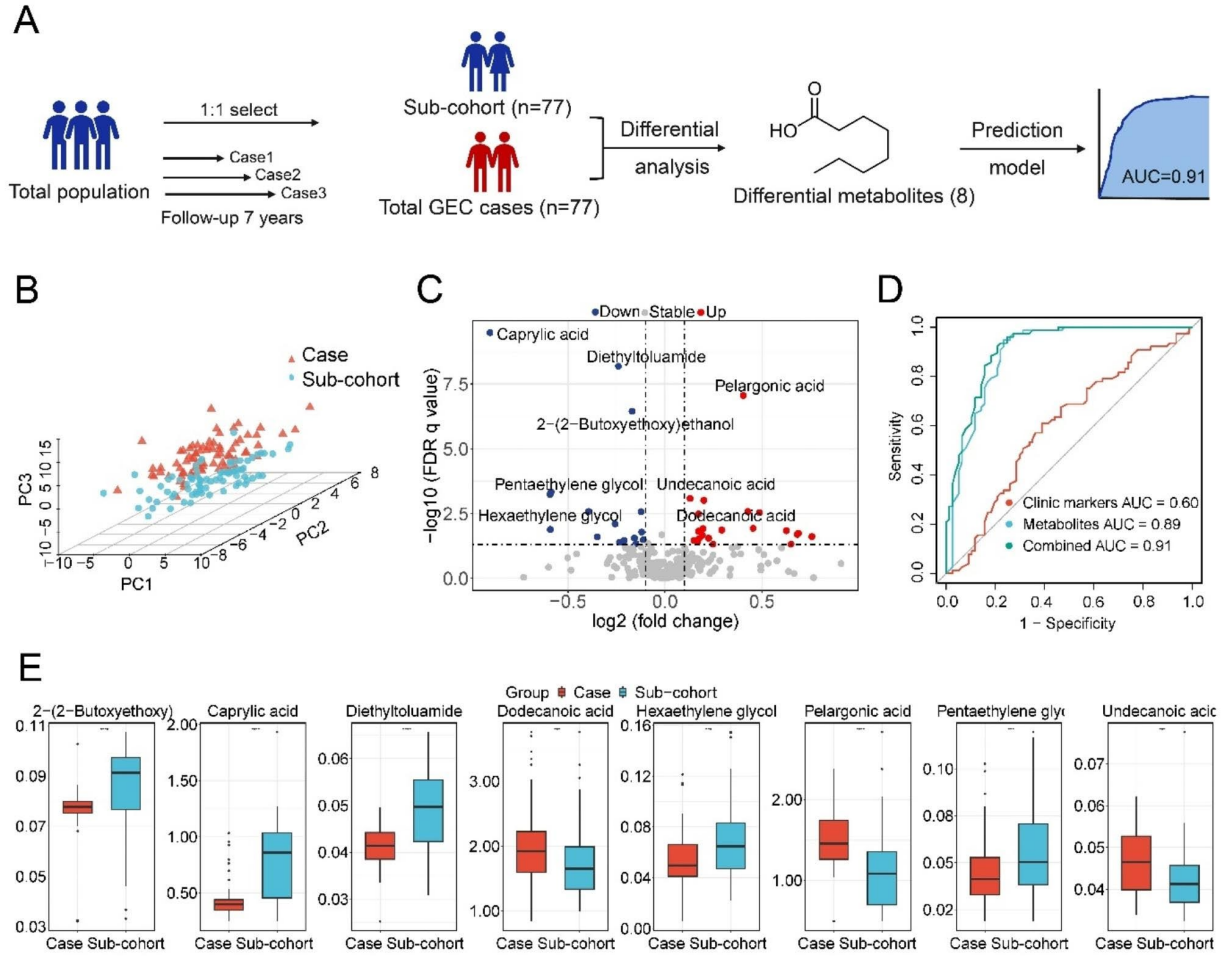
Differential metabolites used to predict progression of GEC in the case-cohort study. **A** The case-cohort study design; **B** PLS-DA three-dimensional scores plot discriminating GEC cases and sub-cohort individuals; **C** Volcano plot showing correlations between FDR q value and FC for all metabolites in GEC cases and sub-cohort individuals; **D** ROC curves of prediction models to predict GEC formation; **E** Boxplots showing levels of differential metabolites in serum samples of GEC cases and sub-cohort individuals

Figure 1A shows the study design of our prospective case-cohort study. The initial cohort was followed up until January 2022. During the 7-year follow up period, participants who processed to esophageal TIS (n = 14), ESCC (n = 28), and GC (N = 35) were defined as GEC cases (n = 77). Sub-cohort individuals were randomly selected from the initial cohort in a 1:1 ratio (n = 77).

### Sociodemographic characteristics and lifestyle factors

Self-reported sociodemographic characteristics included age, sex (male/female), body mass index (BMI), education status (lack of education, primary, junior, senior, college or above), marriage status (married/unmarried), number of family members (1, 2, ≥ 3), family medical history (yes/no). Lifestyle factors included smoking status (current or former smokers, vs. non-smokers), alcohol drinking status (current or former drinkers, vs. non-drinkers), tea drinking status (current or former tea drinkers, vs. non-drinkers), dairy intake (never/rarely, sometimes, usually), beans intake (never/rarely, sometimes, usually), salted food intake (never/rarely, sometimes, usually), fried food intake (never/rarely, sometimes, usually), and hot food intake (never/rarely, sometimes, usually).

### Serum collection

All participants were in a state of overnight fasting, and 5 mL of peripheral venous blood was collected in the morning. The serum samples were then allowed to clot for 30 min at 37℃ water batch, followed by centrifugation at 3,000 rpm for 15 min. Subsequently, the serum supernatant was extracted, immediately frozen in liquid nitrogen, and stored at -80℃ until further analyses.

### UPLC-TOF-MS analysis and data preprocessing

Prior to UPLC-TOF-MS analysis, serum samples were thawed at 4℃ on ice and prepared according to the procedure in the methods section of Supplementary Materials. The serum samples were analyzed by UPLC-QTOF/MS using reverse-phase liquid chromatography (RPLC) chromatographic column. The column parameter was set to 2.6 μm, 100 mm (length)× 2.1 mm, Phenomenex, and UHPLC Kinetex C18 chromatographic column. The flow rate was 0.3mL/min, every injection lasted 12 min. The solvent A of the chromatographic mobile phase was water (100%) and 0.01% acetic acid, and the solvent B was acetonitrile (50%) and isopropanol (50%). The gradient elution conditions were as follows: 0–1 min, 99% A and 1% B; 8–9 min, 1% A and 99% B; 10–12 min, 99% A and 1% B.

The mass spectrometry detection equipment was a Thermo Scientific Orbitrap Exploris 480 mass spectrometer, and the ion source was a Thermo Scientific EASY-IC ion source, whose accuracy was controlled below 1 ppm by real-time adjustment of the m/z calibration. The equipment was divided into positive ion mode (POS) and negative ion mode (NEG). The data acquisition mode included MS1 using full scan mode, and MS2 using data-dependent acquisition mode (DDA).

Following UPLC-TOF-MS analysis, the raw data was preprocessed and annotated (see details in the methods section of Supplementary Materials). Finally, 305 metabolites in the case-cohort study were identified for subsequent analysis.

### Statistical analysis

Partial least squares discrimination analysis (PLS-DA) was applied to explore the separation tendency of metabolic profile between cases and sub-cohort group. The goodness of fit for the PLS-DA models was evaluated by the explained variations (R^2^) and the predicted variations (Q^2^). Wilcoxon rank sum test and PLS-DA were then applied to identify differential metabolites for discriminating GEC pathological progression individuals from sub-cohort. Metabolites with *P* value < 0.05 were defined as significantly altered metabolites, and with FDR q value < 0.05 and variable importance of the projection (VIP) > 1 were viewed as differential metabolites in this study.

Three random forest (RF) models were developed to evaluate the predictive value of the differential metabolites for GEC progression, including a clinical markers model, a metabolites model, and a combined model (incorporating both clinical markers and metabolites). These models were then assessed using leave-one-out cross-validation (LOOCV). Furthermore, we utilized the ESCC screening cohort (n = 1104) to assess the predictive performance of these specific GEC progression-related metabolites for the early screening of ESCC. Participants were randomly divided into a discovery set (n = 662) and a validation set (n = 442). The detailed materials can be found in our previous publication [14]. The area under the receiver operating characteristic curve (AUC), net reclassification index (NRI), and integrated discrimination improvement (IDI) were calculated to evaluate the predictive performance.

Pathway enrichment analysis of differential metabolites was conducted using MetaboAnalyst (https://www.metaboanalyst.ca/) based on the Kyoto Encyclopedia of Genes and Genomes (KEGG) pathway database [19]. All analyses were conducted using the R platform (version 4.3.0).

## Results

### Participant characteristics

Table 1 presents the baseline characteristics of participants in the case-cohort study. This study encompasses 154 participants (mean [SD] age, 58.7 [7.1] years; 30.5% female; mean [SD] BMI, 23.8 [3.1] kg/m^2^), of which 14 progressed to TIS, 28 progressed to ESCC and 35 progressed to GC.

**Table 1:** Baseline characteristics of the case-cohort study population

Variable	Sub-cohort (n = 77)	Case (n = 77)	Total (n = 154)	*P* value
**Demographic**				
Age (years)	57.7 (7.6)	59.8 (6.4)	58.7 (7.1)	0.068
Female, n (%)	29 (37.7)	18 (23.4)	47 (30.5)	0.080
BMI (kg/m^2^)	24.1 (2.9)	23.5 (3.3)	23.8 (3.1)	0.228
Education, n (%)				0.380
Lack of education	10 (13.0)	10 (13.0)	20 (13.0)	
Primary	18 (23.4)	27 (35.1)	45 (29.2)	
Junior	41 (53.2)	31 (40.3)	72 (46.8)	
Senior	8 (10.4)	8 (10.4)	16 (10.4)	
College or above	0 (0.0)	1 (1.3)	1 (0.6)	
Married, n (%)	75 (97.4)	72 (93.5)	147 (95.5)	0.155
No. of family members, n (%)				0.701
1	2 (2.6)	4 (5.2)	6 (3.9)	
2	27 (35.1)	27 (35.1)	54 (35.1)	
≥ 3	48 (62.3)	46 (59.7)	94 (61.0)	
Family medical history, n (%)	12 (15.6)	14 (18.2)	26 (16.9)	0.827
**Lifestyle**				
Smokers, n (%)	21 (27.3)	27 (35.1)	48 (31.2)	0.380
Alcohol drinkers, n (%)	23 (29.9)	28 (36.4)	51 (33.1)	0.490
Tea drinkers, n (%)	50 (64.9)	50 (64.9)	100 (64.9)	1.000
Dairy intake, n (%)				0.779
Never/rarely	0 (0.0)	0 (0.0)	0 (0.0)	
Sometimes	8 (10.4)	6 (7.8)	14 (9.1)	
Usually	69 (89.6)	71 (92.2)	140 (90.9)	
Beans intake, n (%)				0.141
Never/rarely	0 (0.0)	0 (0.0)	0 (0.0)	
Sometimes	37 (48.1)	27 (35.1)	64 (41.6)	
Usually	40 (51.9)	50 (64.9)	90 (58.4)	
Salted food intake, n (%)				0.873
Never/rarely	2 (2.6)	2 (2.6)	4 (2.6)	
Sometimes	52 (67.5)	49 (63.6)	101 (65.6)	
Usually	23 (29.9)	26 (33.8)	49 (31.8)	
Fried food intake, n (%)				0.970
Never/rarely	1 (1.3)	1 (1.3)	2 (1.3)	
Sometimes	66 (85.7)	67 (87.0)	133 (86.4)	
Usually	10 (13.0)	9 (11.7)	19 (12.3)	
Hot food intake, n (%)				0.076
Never/rarely	44 (57.1)	30 (39.0)	74 (48.1)	
Sometimes	17 (22.1)	23 (29.9)	40 (26.0)	
Usually	16 (20.8)	24 (31.2)	40 (26.0)	

Data are means ± SD, or n (%). BMI = Body mass index

**Fig. 2:**
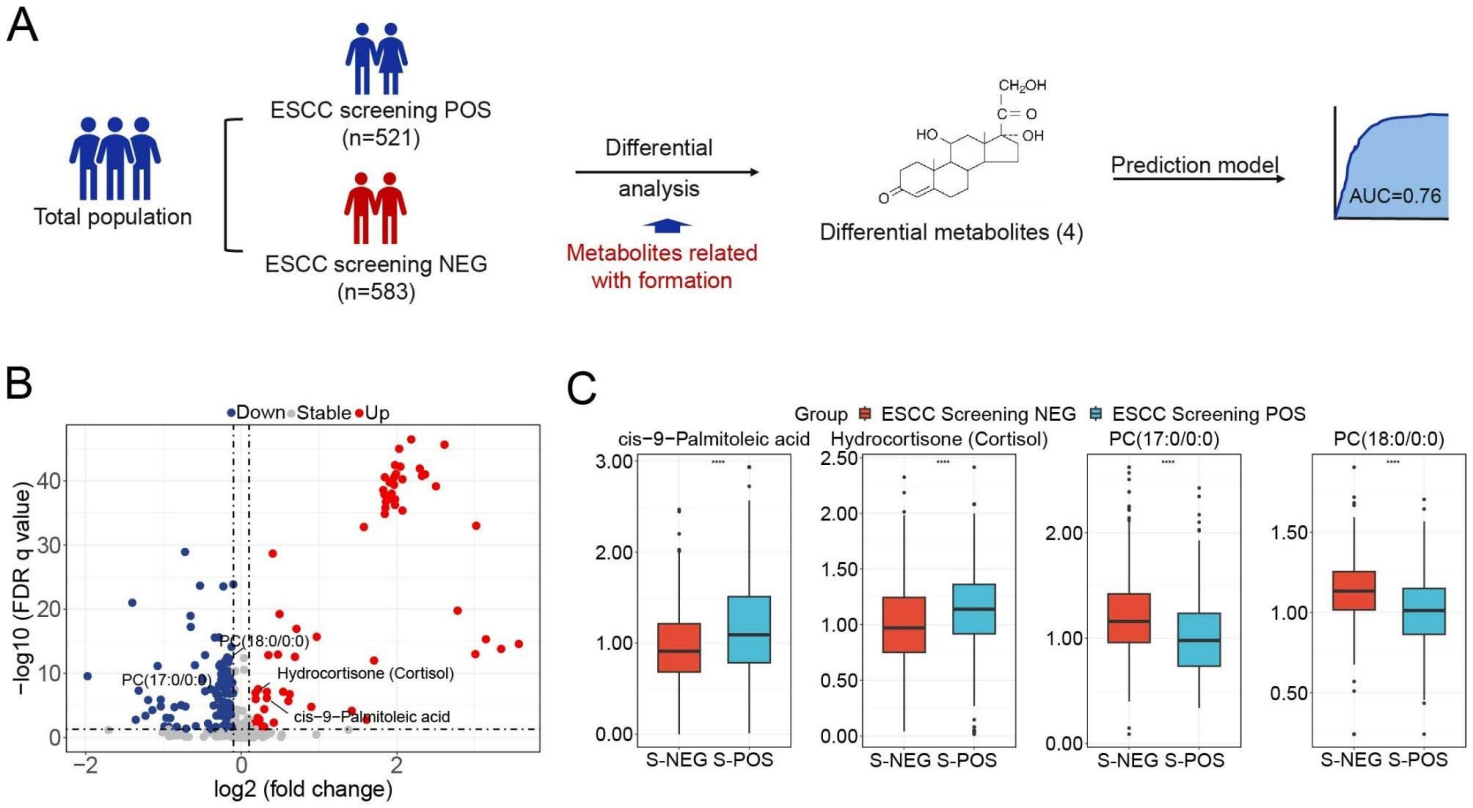
Differential metabolites associated with progression of GEC in the screening cohort; **A** The screening study design; **B** Volcano plot showing correlations between FDR q value and FC for all metabolites in ESCC screening-positive and screening-negative group;  **C**  Boxplots showing levels of differential metabolites in serum samples of screening-positive and screening-negative group

In the screening study, we enrolled 1104 participants (discovery set, mean [SD] age, 56.1 [7.9] years, 53.6% female) (Fig. 2A). ESCC screening-positive subjects tended to be older and had higher levels of systolic blood pressure and education. Additionally, a greater proportion of these subjects were wellspring drinkers and smokers compared to health controls in the discovery set (Table S1).

### Differential metabolites associated with GEC formation

The PLS-DA score plot demonstrated a distinct metabolic profile separation between GEC cases and sub-cohort individuals (Fig. 1B). As shown in Fig. 1C, univariate analysis identified 41 significantly altered metabolites (*P* value < 0.05), including 22 up-regulated and 19 down-regulated). Of these metabolites, eight were classified as differential metabolites by FDR q value < 0.05 and VIP > 1, to discriminate GEC cases from the sub-cohort group (Table 2, Table S2). Figure 1E showed detailed information on the eight metabolites, where five metabolites were inversely associated with the risk of progression of GEC, including 2-(2-butoxy ethoxy) ethanol, caprylic acid, diethyltoluamide, hexamethylene glycol, and pentaethylene glycol. The other three metabolites (dodecanoic acid, pelargonic acid, and undecanoic acid) were positively associated with the risk of GEC progression.

**Table 2:** Detailed information of 8 differential metabolites identified in the case-cohort study

Metabolites	m/z	RT	*P* value	FDR	FC ^a^	VIP
2-(2-butoxy ethoxy) ethanol	163.133	307.600	< 0.001	< 0.001	0.889	2.464
Caprylic acid	143.108	427.300	< 0.001	< 0.001	0.535	4.311
Diethyltoluamide	192.138	379.700	< 0.001	< 0.001	0.846	3.561
Dodecanoic acid	199.170	511.100	< 0.001	0.037	1.149	1.524
Hexamethylene glycol	283.175	221.900	< 0.001	0.030	0.663	1.823
Pelargonic acid	157.123	452.900	< 0.001	< 0.001	1.323	2.804
Pentaethylene glycol	239.149	213.300	< 0.001	0.028	0.665	1.726
Undecanoic acid	185.155	476.100	< 0.001	0.036	1.094	2.166

m/z = Mass charge ratio, RT = Retention time, FDR = *P* value adjusted using false discovery rate, FC = Fold change, VIP = The variable importance in projection

^a^ Fold change was calculated as the ratio of the mean values of GEC cases to sub-cohort individuals

Furthermore, PLS-DA identified a clear separation tendency between the sub-cohort and the TIS group, the sub-cohort and the ESCC group, and the sub-cohort and the GC group (Figure S2). We found 6 out of 14 differential metabolites in the TIS vs. sub-cohort, 5 out of 7 in the ESCC vs. sub-cohort, and all 4 in the GC vs. sub-cohort were identical to the above 8 metabolites, subsequently (Table S3-5 and Figure S3-5).

**Fig. 3:**
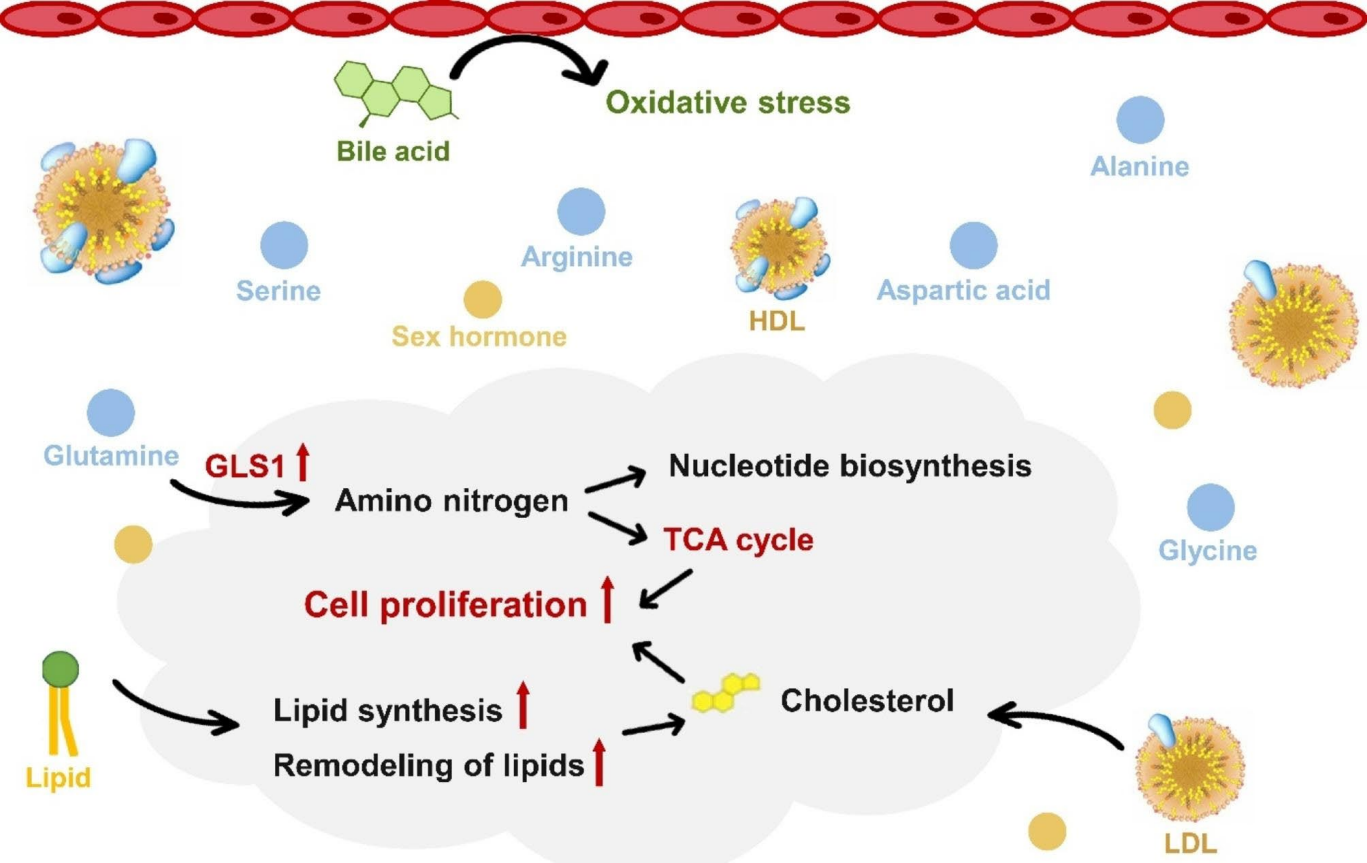
Schematic representation of the effect of differential metabolites on GEC progression

### Prediction model to discriminate GEC formation

Figure 1D demonstrated that clinical markers (age, sex, body mass index [BMI], education, vegetable intake, fruit intake, bean intake, and hot food intake) exhibited poor performance in pathological processes prediction (AUC = 0.599; 95% CI = 0.505–0.683). In contrast, the panel of eight metabolites had a robust AUC value of 0.893 (95% CI = 0.816–0.951), with a high sensitivity of 0.961 (95% CI = 0.896-1.000), specificity of 0.766 (95% CI = 0.662–0.857). The combined RF model, integrating clinical markers and eight metabolites, achieved the best prediction performance (AUC = 0.914; 95% CI = 0.861–0.955). The NRI and IDI for the comparison of the combined model versus the clinical markers-only model were 0.507 (95% CI = 0.332–0.681) and 0.451 (95% CI = 0.370–0.532), respectively. Figure S6 summarizes the predictive performance of the RF models across different pathological progression (TIS, ESCC, and GC), with the combined models for ESCC showing particularly strong predictive performance (AUC = 0.907; 95% CI = 0.849–0.954).

### GEC progression-related metabolites showing promising results for early screening of ESCC

Among the metabolites that were significantly altered between GEC cases and sub-cohort individuals (*P* value < 0.05), 17 metabolites were included in the screening study (Fig. 2B, Table S7). Subsequently, four metabolites, with FDR q value < 0.05 and VIP > 1, were identified as differential metabolites for the early screening of ESCC, of which two were positively associated with ESCC, including cis-9-palmitoleic acid and Hydrocortisone. In contrast, the other two metabolites, PC (17:0/0:0) and PC (18:0/0:0) were found to be inversely related to ESCC (Fig. 2C and Table S7).

Subsequently, we developed a RF model incorporating four differential metabolites for ESCC screening in the discovery set and evaluated this prediction performance in the validation set. Compared to clinical markers-only, the combined model of clinical markers and metabolites achieved an AUC value of 0.810 (95% CI = 0.779–0.843) in the discovery set and 0.761 (95% CI = 0.716–0.805) in the validation set (Table S8, Figure S6). The NRI for the combined model versus the clinical markers-only model was 0.169 (95% CI = 0.087–0.252) in the discovery set and 0.184 (95% CI = 0.071–0.297) in the validation set, and IDI was 0. 097 (95% CI = 0.061, 0.132) and 0.079 (95% CI = 0.032, 0.125), respectively (Table S9).

### Dysregulated metabolic pathways associated with GEC formation

Using significantly altered metabolites, we conducted enrichment analyses, based on the KEGG database, to identify the pathways dysregulated in GEC patients (Table S10). These analyses revealed significant disruption in D-glutamine and D-glutamate metabolism, nitrogen metabolism, primary bile acid biosynthesis, and steroid hormone biosynthesis (Fig. 3). Notably, D-glutamine and D-glutamate metabolism and nitrogen metabolism were also associated with ESCC (Table S11).

## Discussion

GEC is the first leading cause of digestive cancer mortality, which is typically diagnosed and treated at advanced stages [2]. Thus, early detection of GEC provides opportunities to implement effective and timely treatment strategies to enhance patients outcomes. However, there is currently little study on pathological progression from esophagitis, atypical hyperplasia to GEC. In our prospective case-cohort study at the Esophageal Cancer Screening Base in Shandong Province, we observed a distinct separation between GEC cases and sub-cohort individuals and identified eight differential metabolites. Four metabolic pathways were also found to be influenced by metabolic dysregulation in GEC progression. The panel of the GEC pathological progression-related metabolites showed strong predictive performance both in GEC progression and in the early screening of ESCC.

A series of metabolites, including cortisol, lipid, and acid, were found to be significantly altered between GEC cases and sub-cohort individuals, indicating the potential dysregulation of steroid hormone biosynthesis and bile acid biosynthesis pathways during GEC progression. Steroid hormone biosynthesis, including cholesterol synthesis, glucocorticoid synthesis, and sex hormone synthesis, is a critical pathway in cancer development [20]. For instance, CYP19A1, a steroid synthetase, has been implicated in promoting the progression of gastric cancer [21]. Cholesterol-rich lipid droplets and lipoprotein receptors were commonly observed in patients with gastric cancer, among which low-density lipoprotein (LDL) and high-density lipoprotein (HDL) were the main cholesterol carriers [21]. Some studies indicated potential risks of LDL and HDL in the progression of gastric cancer [21, 22]. Relative research confirmed that steroid biosynthesis also played a crucial role in ESCC [18], with lipid biosynthesis and remodeling being active in cancer cells [23].

Bile acids, products of cholesterol metabolism, may induce oxidative stress and reactive oxygen production, leading to inflammation, DNA damage, and alteration in cell proliferation and apoptosis, all of which were susceptibility factors for cancer [24]. The expression of CDX2, an early event in inflammation-to-carcinogenesis progression, is also triggered by bile acid reflux [25]. Recent studies have shown that short-term exposure to bile acids increased esophageal cancer risk [26], and Glycochenodeoxycholic acid (GCDCA) elevation, noted in ESCC patients [27], aligns with our findings.

Furthermore, our study identified dysregulation of glutamate and glutamine in the progression of GEC, which was also evident in the early screening of ESCC. Glutamine, crucial for nitrogen and carbon supply in biosynthesis and an important energy source via TCA cycle, supports cancer cell growth and proliferation [28]. The main rate-limiting enzyme for glutamine catabolism, GLS1, is highly expressed in various cancers, including small-cell lung cancer, hepatic carcinoma, and ESCC, with its down-regulation impacting cancer cell proliferation, invasion, and migration [29]. Elevated glutamate levels [30] and active amino acid metabolism [31] in esophageal cancer patients compared to health controls further corroborate our findings, suggesting glutamine as a sensitive metabolic marker for GEC progression.

This study had important strengths. Firstly, the implementation of a prospective case-cohort study design was resource-efficient, optimizing sample size and conserving manpower and financial resources. Secondly, different from current metabolic research focused primarily on early screening, our study explored metabolites associated with the pathological progression of GEC. Furthermore, sensitivity analyses reinforced the consistency and reliability of our findings. Lastly, we also evaluated the discrimination performance of metabolites related to GEC progression in the early screening of ESCC.

Our study also had several shortcomings. Firstly, as a single-center exploratory study with a relatively modest sample size, the findings from our case-cohort study require validation through external datasets to confirm their reliability. While we have utilized an ESCC screening cohort for validation, broader verification is essential. Secondly, current studies on differential metabolites and their pathways were based on population, and more basic experiments were needed to be verified. Finally, due to the separation of the case-cohort study and screening study into two distinct batches of metabolomics experiments, more precise metabolomics identification methods are needed to enhance the accuracy of metabolite matching.

## Conclusion

In conclusion, our prospective case-cohort study successfully identified differential metabolites between GEC cases and sub-cohort individuals, which had strong predictive performance for GEC pathological progression and for the early screening of ESCC. This finding is crucial for the early screening and timely intervention in GEC high-risk groups, potentially reducing mortality and disease burden. In addition, our study implied that specific metabolites were abnormal during GEC progression, affecting the tumor cell microenvironment and thus leading to the irreversible transformation of early gastroesophageal lesions to GEC. These insights might provide valuable clues for the exploration of GEC pathological mechanism.

### Electronic supplementary material

Below is the link to the electronic supplementary material.

Supplementary Material 1

Supplementary Material 2

Supplementary Material 3

## Data Availability

The data generated in this study are not publicly available due to the privacy laws, but are available upon reasonable request from the corresponding author.

## Abbreviations

GEC: gastroesophageal cancer
ESCC: esophageal squamous cell carcinoma
VIP: variable importance in projection
RF: random forest
AUROC: area under the receiver operating characteristic curve
TIS: tumor in situ
AAG: alanine,aspartate and glutamate
GC: gastric cancer
RPLC: reverse-phase liquid chromatography
POS: positive
NEG: negative
DDA: data-dependent acquisition
PCA: principal component analysis
QC: quality control
PLS-DA: partial least squares discrimination analysis
NRI: net reclassification index
IDI: integrated discrimination improvement
KEGG: Kyoto Encyclopedia of Genes and Genomes
HDL: high-density lipoprotein
LDL: low-density lipoprotein
