# Supplementary material for: Untargeted serum metabolomics reveals potential biomarkers and metabolic pathways associated with the progression of gastroesophageal cancer
Source: BMC Cancer. 2023 Dec 15;23:1238. doi: 10.1186/s12885-023-11744-y (PMC10724912; doi:10.1186/s12885-023-11744-y)
Supplement: Supplementary file 1 — Supplementary Material 1 [file 12885_2023_11744_MOESM1_ESM.pdf]

# Additional file: Supplement Methods

## Inclusion and exclusion criteria of the case-cohort study

After excluding individuals without complete baseline information or those who did not progress beyond mild esophagitis, we established the initial cohort for the case-cohort study (n = 3514; see methods section of the Supplementary Materials and Figure S1). Among these individuals, those who had available serum samples and processed to esophageal TIS (n = 14), ESCC (n = 28), and GC (n = 35) during the 7-year follow up period, were defined as GEC cases (n = 77). Sub-cohort individuals were randomly selected in a 1:1 ratio (n = 77). The inclusion criteria of the case-cohort study were as follows: (1) Baseline pathology of esophagitis, mild, moderate, or severe dysplasia; (2) Availability of complete basic information and biological specimens; (3) Follow-up duration of more than 1 years; (4) Pathological progression to TIS, ESCC, or GC; (5) Absence of any other metabolic disease (such as hyperlipidemia, diabetes, hyperthyroidism, etc.); (6) Absence of any other malignant tumors.

## Diagnostic code

ESCC screening-negative subjects (n=583) were identified as those with normal esophageal mucosa, confirmed positive iodine staining. Conversely, ESCC screening-positive subjects (n=521) were characterized by negative iodine staining results. Biopsies of the iodine-negative participants (ESCC screening-positive subjects) were taken from the non-staining area of the mucosa, which were then underwent pathological evaluation to confirm and stage by two pathologists.

## Sample preparation

To ensure the stability of metabolites, serum samples were thawed at 4°C on ice. The quality control (QC) samples were prepared by mixing equal amounts of each serum sample, and were run four times in randomized order within every analytical batch to monitor the stability of the analysis throughout the whole experimental procedures. In this study, a total of 9 QC samples and 130 QC samples were used in the case-cohort study and the screening study respectively. Then the organic solvent precipitation method was used to remove denatured proteins after precipitation at a low temperature by high-speed centrifugation. The concrete operation was as follows: Firstly, 400μL methanol/acetonitrile (1:1) was added into 100μL serum. Then every serum was vortexed for 30 seconds and sonicated for 10 minutes in a water bath at 4°C, and incubated for 1 hours at -20°C to facilitate protein precipitation. Then it was centrifuged at 13,000 rpm in 4°C for 15 minutes. The serum supernatant was removed and evaporated to dryness using a vacuum concentrator at 4°C prior to UPLC-QTOF/MS analysis.

## Data preprocessing and annotation

The raw metabolic profiling data file obtained by the UPLC-QTOF/MS was composed of a complex three-dimensional data format, including retention times (RT), m/z scores and density or abundance on each axis. Operations such as retention time alignment, data filtering, peak identification and peak extraction of the original data were carried out through the MetFlow website (https://metflow.zhulab.cn/). Then peaks were annotated using MetDNA2 website (https://metdna.zhulab.cn/) to obtain the sample of total ion peaks, and the m/z and RT

of each corresponding ion peak. Peaks with isotopes, weak signals less than 1000, and detection rate of QC samples less than 80% were removed. In the case-cohort study, peaks for which RSD>30% (23 out of 583) were excluded from the analysis, RPLC chromatographic column was selected (347 out of 560), and metabolites with the same m/z and RT were deduplicated (42 out of 347). In the screening study, peaks for which RSD>30% (337 out of 8182 in discovery set, 889 out of 8182 in validation set), were excluded from the analysis. Unnamed metabolites (7442 out of 7845 in the discovery set, 6880 out of 7293 in the validation set) were excluded. Finally, 305 metabolites in the case-cohort study and 403 metabolites in the screening study were selected for subsequent analysis.
