# Supplementary material for: Untargeted serum metabolomics reveals potential biomarkers and metabolic pathways associated with the progression of gastroesophageal cancer
Source: BMC Cancer. 2023 Dec 15;23:1238. doi: 10.1186/s12885-023-11744-y (PMC10724912; doi:10.1186/s12885-023-11744-y)
Supplement: Supplementary file 2 — Supplementary Material 2 [file 12885_2023_11744_MOESM2_ESM.pdf]

Additional file

Table S1. Baseline characteristics of discovery and validation dataset in the screening study.

Table S2. The detailed information of metabolites which were significantly altered between GEC cases and sub-cohort individuals in case-cohort study.

Table S3. Detailed information of differential metabolites identified in the subgroup case-cohort study (TIS).

Table S4. Detailed information of differential metabolites identified in the subgroup case-cohort study (ESCC).

Table S5. Detailed information of differential metabolites identified in the subgroup case-cohort study (GC).

Table S6. ROC analysis of prediction model to discriminate GEC.

Table S7. Detailed information of metabolites which were significantly altered between ESCC screening positive and ESCC screening negative group identified in the screening study.

Table S8. ROC analysis of prediction model to predict ESCC screening-positive subjects.

Table S9. NRI and IDI values for RF models composed of clinic markers and selected metabolites.

Table S10. Pathway enrichment analysis of differential metabolites in the case-cohort study and in the screening study.

Table S11. Pathway enrichment analysis of differential metabolites in the subgroup case-cohort study.

Table S1. Baseline characteristics of discovery and validation dataset in the screening study

Variable	Discovery dataset (n=662)			Validation dataset (n=442)		
	Screening-NEG (n=311)	Screening-POS (n=351)	*P* value	Screening-NEG (n=272)	Screening-POS (n=170)	*P* value
**Demographic**						
Female, n (%)	179 (57.6)	176 (50.1)	0.067	169 (62.1)	85 (50.0)	0.016
Age (years)	53 (7.6)	58.9 (7.2)	<0.001	53.3 (9.4)	56.7 (12.1)	0.001
Height (cm)	161.4 (7.5)	162.4 (9.5)	0.145	161 (7.1)	163.4 (7)	<0.001
Weight (kg)	64.6 (10.3)	63.1 (9.6)	0.068	63.6 (9.8)	63.3 (9.8)	0.753
BMI (kg/m^2)	24.8 (3.4)	24.3 (7.6)	0.291	24.5 (3.4)	23.7 (3.1)	0.007
SBP (mmHg)	129.8 (24)	134.4 (21.5)	0.009	130.8 (18.6)	133.8 (22.8)	0.133
DBP (mmHg)	84.1 (13.8)	84.3 (11.9)	0.900	83 (10.4)	84.2 (11.7)	0.261
Marriage, n (%)			0.012			0.311
Unmarried	1 (0.3)	2 (0.6)		1 (0.4)	0 (0.0)	
Married	302 (97.1)	320 (91.2)		265 (97.4)	162 (95.3)	
Divorced	0 (0.0)	3 (0.9)		0 (0.0)	1 (0.6)	
Widowed	8 (2.6)	26 (7.4)		6 (2.2)	7 (4.1)	
Education, n (%)			<0.001			0.209
Lack of education	27 (8.7)	61 (17.4)		29 (10.7)	30 (17.6)	
Primary	85 (27.3)	130 (37.0)		94 (34.6)	60 (35.3)	
Junior	172 (55.3)	127 (36.2)		121 (44.5)	61 (35.9)	
Senior	27 (8.7)	32 (9.1)		26 (9.6)	18 (10.6)	
College or above	0 (0.0)	1 (0.3)		2 (0.7)	1 (0.6)	
**Lifestyle**						
Water source, n (%)			<0.001			0.059
Cellar	0 (0.0)	0 (0.0)		3 (1.1)	5 (2.9)	

Lake and river	0 (0.0)	0 (0.0)		0 (0.0)	1 (0.6)	
Well spring	82 (26.4)	317 (90.3)		251 (92.3)	144 (84.7)	
Tap water	229 (73.6)	34 (9.7)		18 (6.6)	20 (11.8)	
Smokers, n (%)	54 (17.4)	94 (26.8)	0.005	21 (23.9)	33 (29.7)	0.445
Alcohol drinkers, n (%)	76 (24.4)	110 (31.3)	0.059	30 (34.1)	40 (36.0)	0.892
**Pathology**						
Esophagitis, n (%)	-	78 (22.2)	-	-	56 (32.9)	-
Mild dysplasia, n (%)	-	189 (53.8)	-	-	68 (40.0)	-
Moderate dysplasia, n (%)	-	41 (11.7)	-	-	33 (23.6)	-
Severe dysplasia, n (%)	-	15 (4.3)	-	-	5 (2.9)	-
TIS, n (%)	-	12 (3.4)	-	-	4 (2.4)	-
Invasive tumor, n (%)	-	16 (4.6)	-	-	4 (2.4)	-

Data are means ± SD, or n (%). BMI = Body mass index, SBP = Systolic blood pressure, DBP = Diastolic blood pressure.

Table S2. The detailed information of metabolites which were significantly altered between GEC cases and sub-cohort individuals in case-cohort study.

Compound name	m/z	rt	*P* value	FC	VIP
2,4-Dihydroxybutanoic acid	101.024	51.500	0.014	1.225	1.211
Benzoic acid	121.030	324.400	0.021	0.989	1.035
Glutamate	130.050	46.800	0.003	0.918	1.715
Caprylic acid	143.108	427.300	< 0.001	0.535	4.311
2-Aminoadipic acid	144.066	64.400	0.028	1.169	1.266
Pelargonic acid	157.123	452.900	< 0.001	1.323	2.804
2-(2-butoxy ethoxy) ethanol	163.133	307.600	< 0.001	0.889	2.464
2-Biphenylol	169.066	397.300	0.042	0.072	1.027
Undecanoic acid	185.155	476.100	< 0.001	1.094	2.166
Diethyltoluamide	192.138	379.700	< 0.001	0.846	3.561
Dodecanoic acid	199.170	511.100	< 0.001	1.149	1.524
Indoxyl sulfate	212.002	305.900	0.048	1.187	1.008
12-Methyltridecanoic?acid	227.201	541.400	0.012	1.146	1.699
Pentaethylene glycol	239.149	213.300	< 0.001	0.665	1.726
cis-9-Palmitoleic acid	253.217	545.200	0.034	1.109	1.432
Homovanillic acid sulfate	261.007	263.200	0.048	1.570	1.638
Hexamethylene glycol	283.175	221.900	< 0.001	0.663	1.823
N4-Acetylcytidine	284.089	201.500	0.022	1.143	1.328
Arachidonic acid (AA)	303.233	544.400	0.032	0.926	1.227
trans-11-Eicosenoic acid	309.280	586.400	0.015	1.127	1.784
Adenosine 3',5'-cyclic phosphate (cAMP)	328.045	218.200	0.012	1.371	1.231
Trp-Glu	332.125	196.100	0.013	0.664	1.399
Cortexolone	347.222	365.800	0.003	1.402	1.346
Cortisol	363.217	342.600	0.038	1.124	1.242
21-Deoxycortisol	405.228	365.500	0.003	1.345	1.289

Nonaethylene glycol	415.254	239.800	0.025	0.785	1.441
5beta-Dihydrocortisone	421.223	342.300	0.029	1.130	1.301
Glycochenodeoxycholic acid	448.307	470.200	0.014	1.545	0.784
Glycodeoxycholic acid	448.307	486.000	0.024	1.692	1.039
Glycocholic acid	464.302	431.800	0.02	1.605	0.765
Taurochenodeoxycholic acid	498.289	568.500	0.018	1.612	0.731
LPC(17:2)_RT481	506.324	478.400	0.04	0.850	1.673
LPC(17:1)_RT503	508.340	500.600	0.046	0.901	1.361
LPC(17:0/0:0)	510.355	525.600	0.028	0.896	1.651
Taurocholic acid	514.284	514.800	0.03	2.138	0.962
LPC(18:3)_RT477	518.324	474.400	0.035	0.864	1.281
LPC(18:0/0:0)	524.371	539.500	0.045	0.942	1.219
LPC(19:0)_RT555	538.387	552.200	0.008	0.837	1.580
LPC(22:6)_RT487	568.340	485.100	0.016	0.920	1.291
PC(22:2)_RT521	590.381	518.300	0.003	0.761	1.484
PC(38:4)_RT563	810.600	566.200	0.003	1.128	1.642

m/z = mass charge ratio, RT = Retention time, FC = Fold change, VIP = The variable importance in projection.
Fold change was calculated as the ratio of the mean values of the case group to the sub-cohort group.

Table S3. Detailed information of 14 differential metabolites identified in the subgroup case-cohort study (TIS).

Metabolites	m/z	RT	*P* value	FC	VIP
Caprylic acid[1]	143.108	427.300	<0.001	0.530	2.647
Pelargonic acid[1]	157.123	452.900	0.01	1.237	1.423
2-(2-butoxy ethoxy) ethanol[1]	163.133	307.600	0.001	0.878	1.808
Diethyltoluamide[1]	192.138	379.700	<0.001	0.829	2.475
Pentaethylene glycol[1]	239.149	213.300	0.038	0.677	1.189
Hexamethylene glycol[1]	283.175	221.900	0.037	0.667	1.315
Benzoic acid	121.030	324.400	0.013	0.977	1.487
Glutamate	130.050	46.800	0.042	0.909	1.496
3-Indoleacrylic acid	170.060	344.000	0.036	0.510	1.537
N-[3-(2-oxopyrrolidin-1-yl)propyl]acetamide	185.129	208.600	0.049	1.192	1.384
3alpha-Hydroxy-6-oxo-5alpha-cholan-24-oic acid	389.270	424.800	0.046	1.488	1.973
21-Deoxycortisol	405.228	365.500	0.037	1.415	1.189
PC(30:0)_RT638	706.539	637.100	0.035	1.357	1.369
PC(32:1)_RT639	732.554	637.200	0.025	1.287	1.456

TIS = Tumor in situ, m/z = mass charge ratio, RT = Retention time, FDR = P value adjusted using False Discovery Rate, FC = Fold change, VIP = The variable importance in projection.

Fold change was calculated as the ratio of the mean values of the screening-positive group to the screening-negative group.

The criteria for differential metabolites was *P* value < 0.05 and VIP > 1.

I: Common differential metabolites in the case-cohort study and the subgroup case-cohort study (TIS).

Table S4. Detailed information of 7 differential metabolites identified in the subgroup case-cohort study (ESCC).

Metabolites	m/z	RT	*P* value	FDR	FC	VIP
Caprylic acid [I]	143.108	427.300	< 0.001	< 0.001	0.515	2.932
Pelargonic acid [I]	157.123	452.900	< 0.001	0.011	1.331	2.003
2-(2-butoxy ethoxy) ethanol [I]	163.133	307.600	< 0.001	0.017	0.892	1.635
Diethyltoluamide [I]	192.138	379.700	< 0.001	0.01	0.859	2.204
Dodecanoic acid [I]	199.170	511.100	< 0.001	0.017	1.200	1.446
LPC(18:0/0:0)	524.371	539.500	< 0.001	0.014	0.837	2.101
PC(38:4)_RT563	810.600	566.200	< 0.001	0.017	1.217	2.169

ESCC = Esophageal squamous cell carcinoma, m/z = mass charge ratio, RT = Retention time, FDR = P value adjusted using False Discovery Rate, FC = Fold change, VIP = The variable importance in projection.

Fold change was calculated as the ratio of the mean values of the screening-positive group to the screening-negative group.

The criteria for differential metabolites was FDR q value < 0.05 and VIP > 1.

I: Common differential metabolites in the case-cohort study and the subgroup case-cohort study (ESCC).

Table S5. Detailed information of 4 differential metabolites identified in the subgroup case-cohort study (GC).

Metabolites	m/z	RT	*P* value	FDR	FC	VIP
Caprylic acid[1]	143.108	427.300	< 0.001	< 0.001	0.552	3.487
Pelargonic acid[1]	157.123	452.900	< 0.001	< 0.001	1.352	2.564
2-(2-butoxy ethoxy) ethanol[1]	163.133	307.600	< 0.001	0.009	0.891	2.017
Diethyltoluamide[1]	192.138	379.700	< 0.001	< 0.001	0.843	2.984

GC = Gastric cancer, m/z = mass charge ratio, RT = Retention time, FDR = P value adjusted using False Discovery Rate, FC = Fold change, VIP = The variable importance in projection.

Fold change was calculated as the ratio of the mean values of the screening-positive group to the screening-negative group.

The criteria for differential metabolites was FDR q value < 0.05 and VIP > 1.

I: Common differential metabolites in the case-cohort study and the subgroup case-cohort study (GC).

Table S6. ROC analysis of prediction model to discriminate GEC.

Model	N	AUC	Sensitivity	Specificity
**Total**				
Clinic markers [I]	154	0.599 (0.505, 0.683)	0.649 (0.442, 0.922)	0.610 (0.272, 0.792)
Metabolites [II]	154	0.893 (0.830, 0.944)	0.961 (0.896, 1.000)	0.766 (0.662, 0.857)
Combined [III]	154	0.914 (0.861, 0.955)	0.948 (0.844, 1.000)	0.792 (0.688, 0.896)
**TIS**				
Clinic markers [I]	91	0.506 (0.361, 0.649)	0.929 (0.429, 1.000)	0.286 (0.052, 0.754)
Metabolites [II]	91	0.893 (0.816, 0.951)	1.000 (0.786, 1.000)	0.766 (0.610, 0.948)
Combined [III]	91	0.885 (0.810, 0.948)	1.000 (0.857, 1.000)	0.805 (0.623, 0.909)
**ESCC**				
Clinic markers [I]	105	0.663 (0.542, 0.776)	0.679 (0.429, 0.929)	0.727 (0.325, 0.870)
Metabolites [II]	105	0.902 (0.836, 0.954)	0.929 (0.750, 1.000)	0.844 (0.636, 0.948)
Combined [III]	105	0.907 (0.849, 0.954)	0.929 (0.786, 1.000)	0.792 (0.623, 0.935)
**GC**				
Clinic markers [I]	112	0.538 (0.424, 0.653)	0.714 (0.114, 1.000)	0.481 (0.104, 0.987)
Metabolites [II]	112	0.870 (0.799, 0.925)	0.914 (0.714, 1.000)	0.740 (0.597, 0.922)
Combined [III]	112	0.887 (0.827, 0.942)	0.857 (0.714, 0.971)	0.831 (0.649, 0.935)

AUC = Area under the curve, ESCC = esophageal squamous cell carcinoma, CIS = Carcinoma in situ, GC = Gastric carcinoma.

I: Clinic markers model included age, sex, body mass index, education, vegetable intake, fruit intake, bean intake and hot food intake.

II: Metabolites model included caprylic acid, pelargonic acid, 2-(2-Butoxyethoxy) ethanol, undecanoic acid, diethyltoluamide, dodecanoic acid, pentaethylene glycol, hexaethylene glycol.

III: Combined model included clinic markers and metabolites above.

Area under the curve, sensitivity and specificity of modes were assessed using leave-one-out cross validation (LOOCV).

Table S7. Detailed information of 17 metabolites which were significantly altered between ESCC screening positive and ESCC screening negative group identified in the screening study.

Metabolites	m/z	RT	*P* value	FDR	FC	VIP
**cis-9-Palmitoleic acid**	**253.217**	**498.605**	**< 0.001**	**< 0.001**	**1.256**	**1.204**
**Hydrocortisone (Cortisol)**	**363.216**	**281.484**	**< 0.001**	**< 0.001**	**1.135**	**1.126**
**LPC(17:0/0:0)**	**510.355**	**444.684**	**< 0.001**	**< 0.001**	**0.836**	**1.172**
**LPC(18:0/0:0)**	**524.370**	**472.508**	**< 0.001**	**< 0.001**	**0.898**	**1.316**
2-Ketobutyric acid	101.024	30.038	0.026	0.055	1.026	0.148
L-Pyroglutamic acid	130.050	38.481	0.745	0.81	1.005	0.538
L-Pyroglutamic acid	130.050	66.275	0.704	0.78	1.015	0.559
Dodecanoic acid	199.171	440.017	0.009	0.021	1.131	0.639
Guanosine	284.099	141.771	< 0.001	< 0.001	0.762	0.832
Arachidonic Acid (peroxide free)	303.233	501.496	0.459	0.574	1.079	0.469
PC(18:3/0:0)	518.324	380.537	0.002	0.005	3.050	0.962
PC(22:6/0:0)	568.339	400.680	0.127	0.217	0.972	0.603
PC(14:0/24:4)	810.598	407.118	0.018	0.04	1.212	0.619
PC(14:0/24:4)	810.599	441.457	0.956	0.97	0.952	0.210
PC(14:0/24:4)	810.599	460.072	0.014	0.033	0.872	0.564
PC(14:0/24:4)	810.598	508.986	0.549	0.659	0.959	0.301
PC(14:0/24:4)	810.598	644.281	0.656	0.746	1.056	0.341

m/z = mass charge ratio, RT = Retention time, FDR = P value adjusted using False Discovery Rate, FC = Fold change, VIP = The variable importance in projection.

Fold change was calculated as the ratio of the mean values of the screening-positive group to the screening-negative group.

Table S8. ROC analysis of prediction model to predict ESCC screening-positive subjects.

Model	N	AUC	Sensitivity	Specificity
**Discovery (LOOCV)**				
Clinic markers [I]	662	0.692 (0.651, 0.733)	0.670 (0.513, 0.724)	0.656 (0.588, 0.794)
Metabolites [II]	662	0.685 (0.642, 0.725)	0.786 (0.595, 0.903)	0.540 (0.395, 0.733)
Combined [III]	662	0.810 (0.779, 0.843)	0.772 (0.641, 0.858)	0.746 (0.643, 0.865)
**Validation (External)**				
Clinic markers [I]	442	0.641 (0.591, 0.693)	0.641 (0.329, 0.847)	0.610 (0.368, 0.882)
Metabolites [II]	442	0.692 (0.642, 0.745)	0.688 (0.541, 0.776)	0.676 (0.588, 0.794)
Combined [III]	442	0.761 (0.716, 0.805)	0.729 (0.647, 0.835)	0.728 (0.603, 0.794)

ROC = Receiver operator characteristic curve, ESCC = Esophageal squamous cell carcinoma, AUC = Area under the curve, LOOCV = Leave-one-out cross validation.'

I: Clinic markers model included age, sex, body mass index, education, marriage status, blood pressure, smoke status and alcohol drinking status.

II: Metabolites model included cis-9-Palmitoleic acid, Hydrocortisone (Cortisol), PC(17:0/0:0), PC(18:0/0:0).

III: Combined model included clinic markers and metabolites above.

Table S9. NRI and IDI values for RF models composed of clinic markers and selected metabolites.

	NRI (95% CI)	IDI (95% CI)
**Case-cohort study**		
Total model	0.507 (0.332, 0.681)	0.451 (0.370, 0.532)
TIS model	0.162 (-0.058. 0.383)	0.280 (0.205, 0.355)
ESCC model	0.315 (0.042, 0.588)	0.301 (0.177, 0.425)
GC model	0.496 (0.298, 0.694)	0.250 (0.177, 0.323)
**Screening study**		
Discovery model	0.169 (0.087, 0.252)	0.097 (0.061, 0.132)
Validation model	0.184 (0.071, 0.297)	0.079 (0.032, 0.125)

NRI = Net reclassification improvement, IDI = Integrated discrimination improvement, RF = Random forest, CI = Confidence interval, TIS = Tumor in situ, ESCC = esophageal squamous cell carcinoma, GC = Gastric cancer

Table S10. Pathway enrichment analysis of differential metabolites in the case-cohort study and in the screening study.

Pathways	Total	Expected	Hits	*P* value
**Common pathways**				
D-Glutamine and D-glutamate metabolism	6	0.0273	1	0.027
Nitrogen metabolism	6	0.0273	1	0.027
**Case-cohort study unique pathways**				
Steroid hormone biosynthesis	85	0.664	5	<0.001
Primary bile acid biosynthesis	46	0.359	3	0.005
**Screening study unique pathways**				
Aminoacyl-tRNA biosynthesis	48	0.219	4	<0.001
Phenylalanine, tyrosine and tryptophan biosynthesis	4	0.0182	2	<0.001
Phenylalanine metabolism	10	0.0456	2	<0.001
Biosynthesis of unsaturated fatty acids	36	0.164	2	0.010
Linoleic acid metabolism	5	0.0228	1	0.023
Ubiquinone and other terpenoid-quinone biosynthesis	9	0.041	1	0.040
Caffeine metabolism	10	0.0456	1	0.045

Total means total number of metabolites involved in the specific pathway in Kyoto Encyclopedia of Genes and Genomes (KEGG) dataset; Expected means expected enrichment values of KEGG metabolites in the specific pathway; Hits means the number of differential metabolites enriched in the specific pathway.

Table S11. Pathway enrichment analysis of differential metabolites in the subgroup case-cohort study.

Pathways	Total	Expected	Hits	*P* value
**Common pathways**				
D-Glutamine and D-glutamate metabolism	6	0.0469	1	0.046
Nitrogen metabolism	6	0.0469	1	0.046
**Total case and sub-cohort (unique pathways)**				
Steroid hormone biosynthesis	85	0.664	5	<0.001
Primary bile acid biosynthesis	46	0.359	3	0.005
**ESCC and sub-cohort (unique pathways)**				
Phenylalanine, tyrosine and tryptophan biosynthesis	4	0.013	1	0.013
Phenylalanine metabolism	10	0.0326	1	0.032
Arginine biosynthesis	14	0.0456	1	0.045
Butanoate metabolism	15	0.0488	1	0.048
**CIS and sub-cohort (unique pathways)**				
Arginine biosynthesis	14	0.0182	1	0.018
Butanoate metabolism	15	0.0195	1	0.019
Histidine metabolism	16	0.0208	1	0.021
Alanine, aspartate and glutamate metabolism	28	0.0365	1	0.036
Glutathione metabolism	28	0.0365	1	0.036
Porphyrin and chlorophyll metabolism	30	0.0391	1	0.039
Glyoxylate and dicarboxylate metabolism	32	0.0417	1	0.041
Arginine and proline metabolism	38	0.0495	1	0.049
**GC and sub-cohort (unique pathways)**				
Steroid hormone biosynthesis	85	0.553	5	<0.001
Primary bile acid biosynthesis	46	0.299	3	0.003

Total means total number of metabolites involved in the specific pathway in Kyoto Encyclopedia of Genes and Genomes (KEGG) dataset; Expected means expected enrichment values of KEGG metabolites in the specific pathway; Hits means the number of differential metabolites enriched in the specific pathway.
