# Supplementary material for: Untargeted serum metabolomics reveals potential biomarkers and metabolic pathways associated with the progression of gastroesophageal cancer
Source: BMC Cancer. 2023 Dec 15;23:1238. doi: 10.1186/s12885-023-11744-y (PMC10724912; doi:10.1186/s12885-023-11744-y)
Supplement: Supplementary file 3 — Supplementary Material 3 [file 12885_2023_11744_MOESM3_ESM.pdf]

# Additional file

**Figure S1.** Workflow of population selection of the case-cohort study.

**Figure S2.** Metabolic profile analysis (PLS-DA three-dimensional scores plots and permutation test plots) in the case-cohort study. (A) Sub-cohort and case group profile analysis; (B) Sub-cohort and TIS group profile analysis; (C) Sub-cohort and ESCC group analysis; (D) Sub-cohort and GC group profile analysis.

**Figure S3.** Boxplots showing levels of differential metabolites in serum samples of TIS case group and sub-cohort group.

**Figure S4.** Boxplots showing levels of differential metabolites in serum samples of ESCC case group and sub-cohort group.

**Figure S5.** Boxplots showing levels of differential metabolites in serum samples of GC case group and sub-cohort group.

**Figure S6.** ROC curves of prediction models in the case-cohort study and ESCC screening study. (A) Random forest models to discriminate gastroesophageal cancer; (B) Random forest models to discriminate tumor in situ; (C) Random forest models to discriminate esophageal cancer; (D) Random forest models to discriminate gastric cancer; (E) ROC curves in discovery dataset; (F) ROC curves in validation dataset.

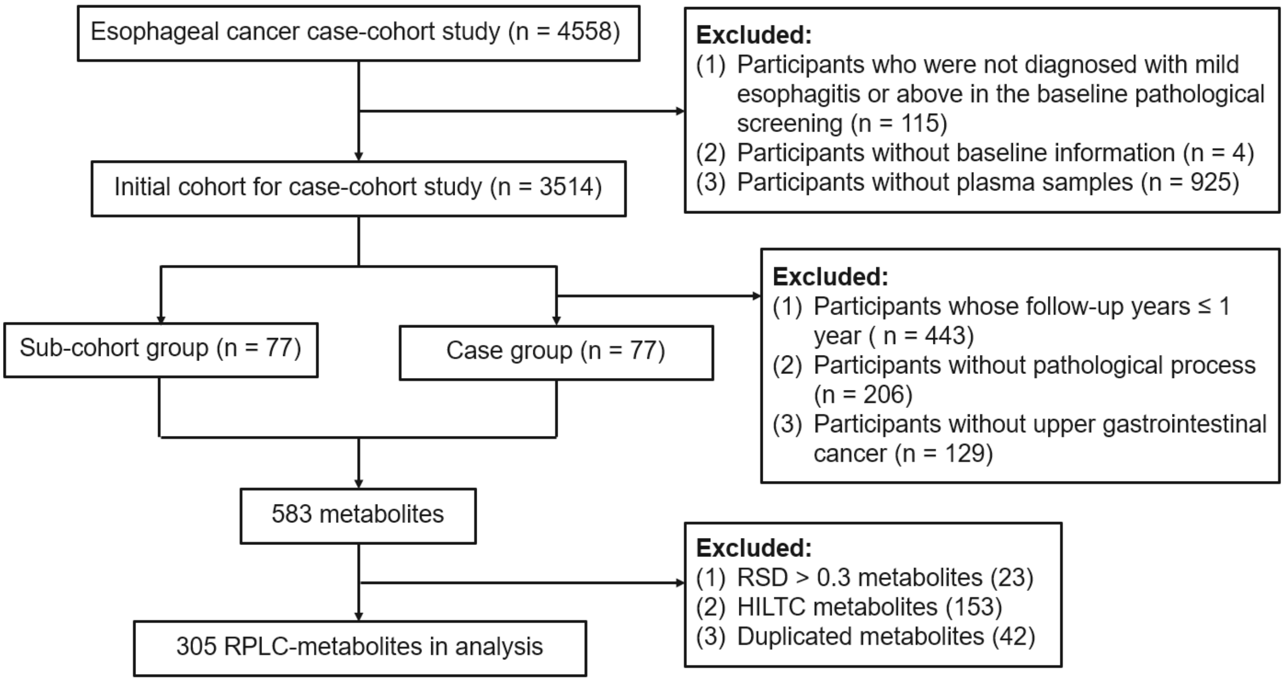


**Figure S1.** Workflow of population selection of the case-cohort study.

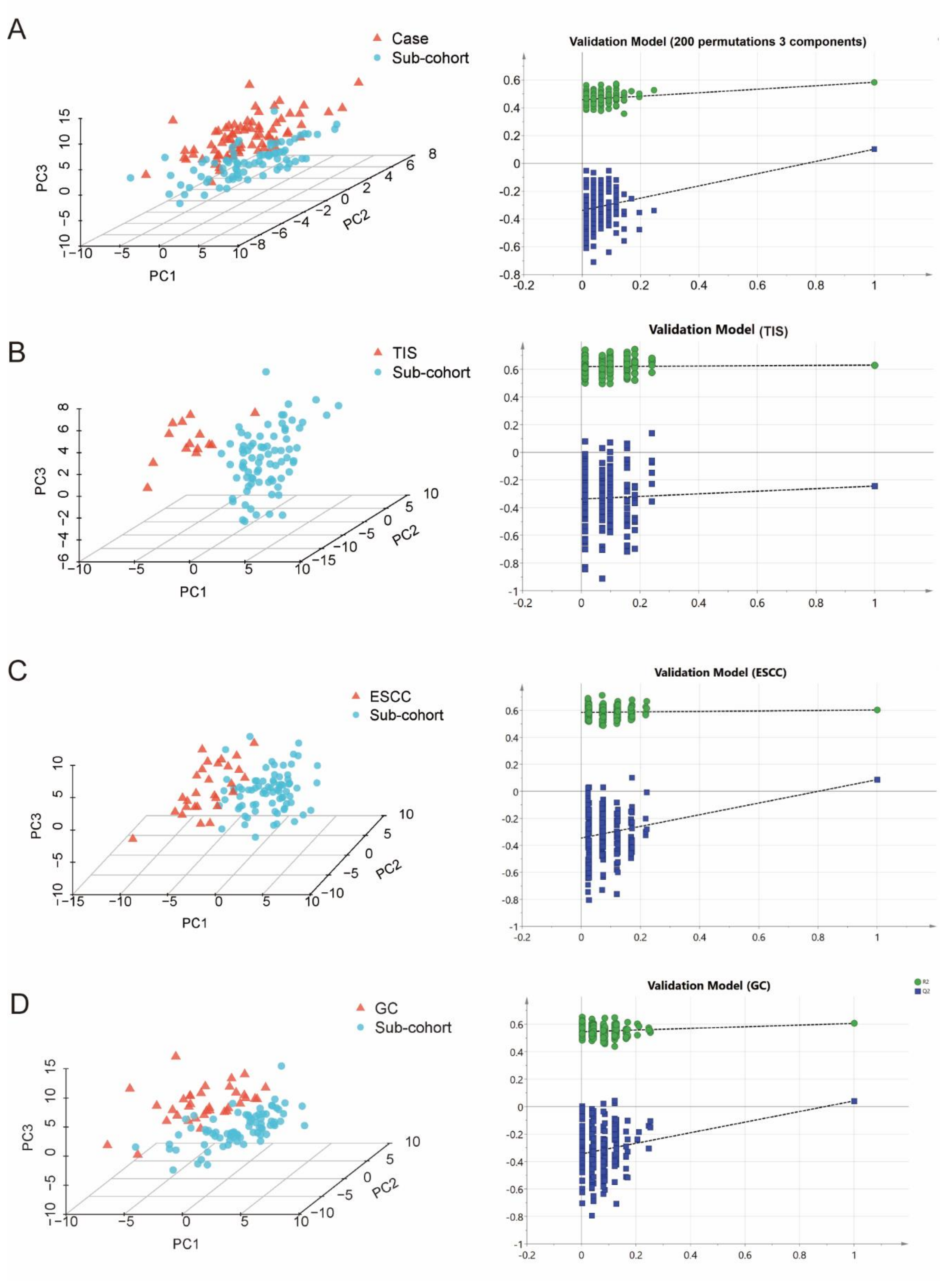


**Figure S2.** Metabolic profile analysis (PLS-DA three-dimensional scores plots and permutation test plots) in the case-cohort study. (A) Sub-cohort and case group profile analysis; (B) Sub-cohort and TIS group profile analysis; (C) Sub-cohort and ESCC group analysis; (D) Sub-cohort and GC group profile analysis.

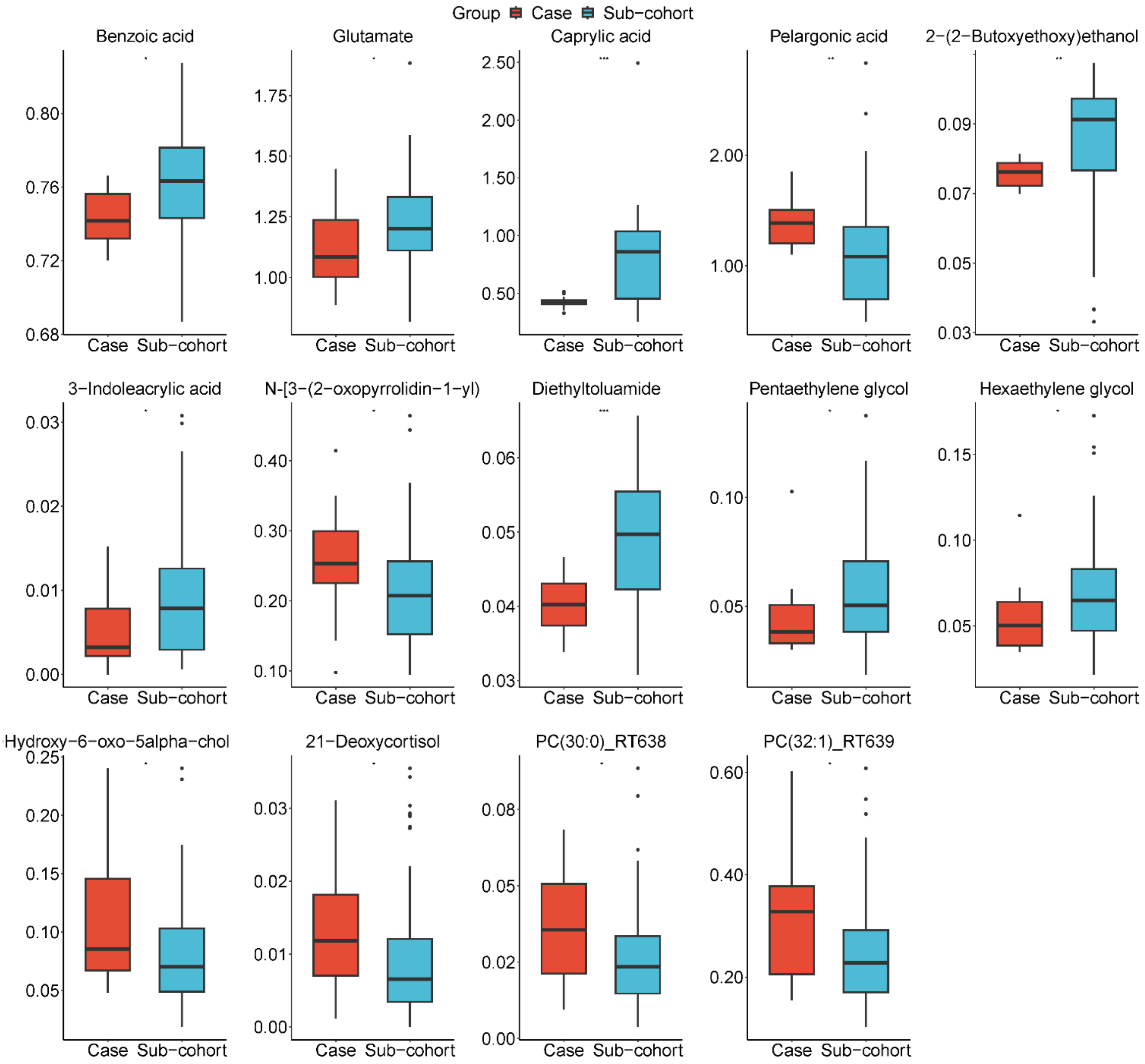


**Figure S3.** Boxplots showing levels of 14 differential metabolites in serum samples of TIS case group and sub-cohort group.

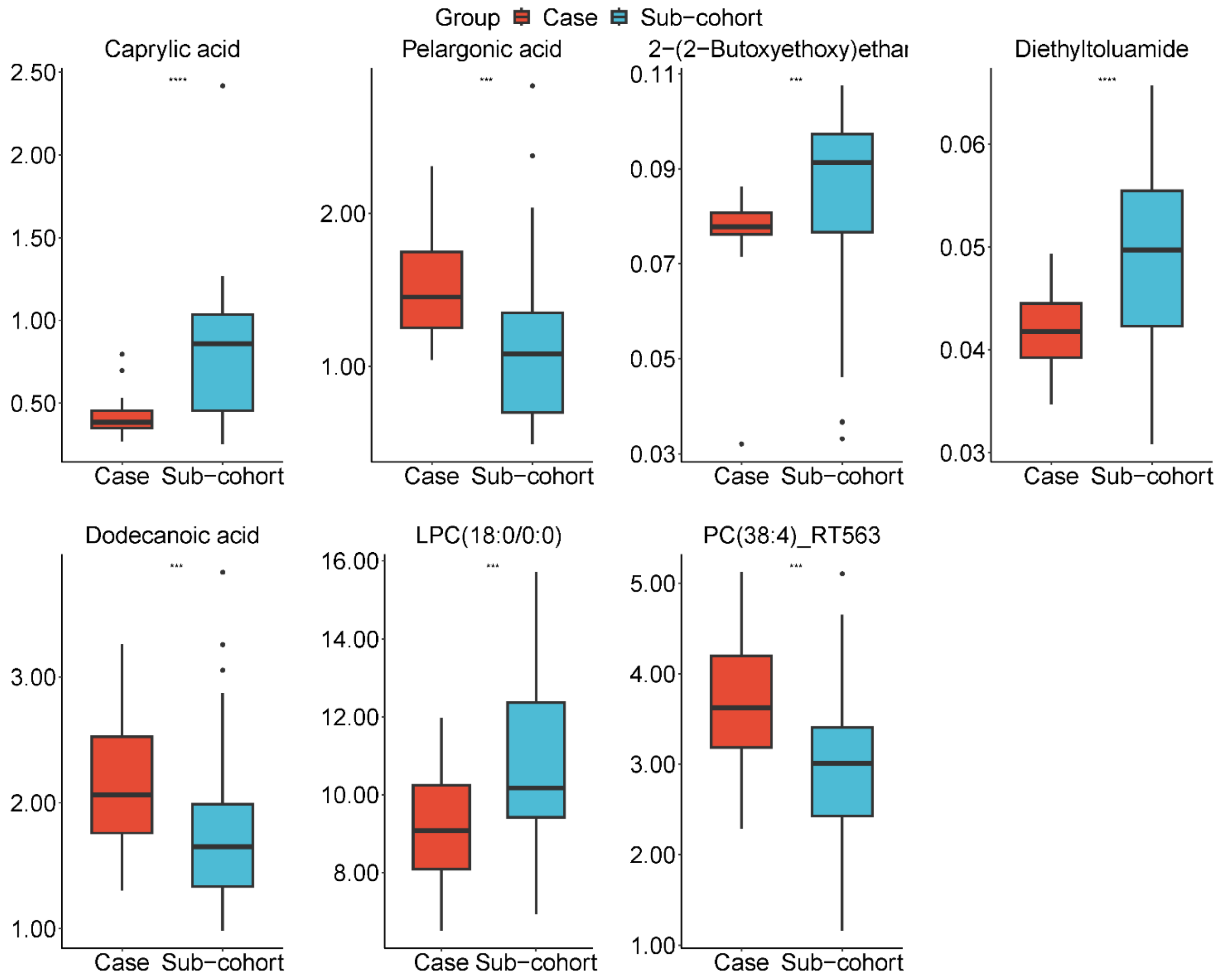


**Figure S4.** Boxplots showing levels of 7 differential metabolites in serum samples of ESCC case group and sub-cohort group.

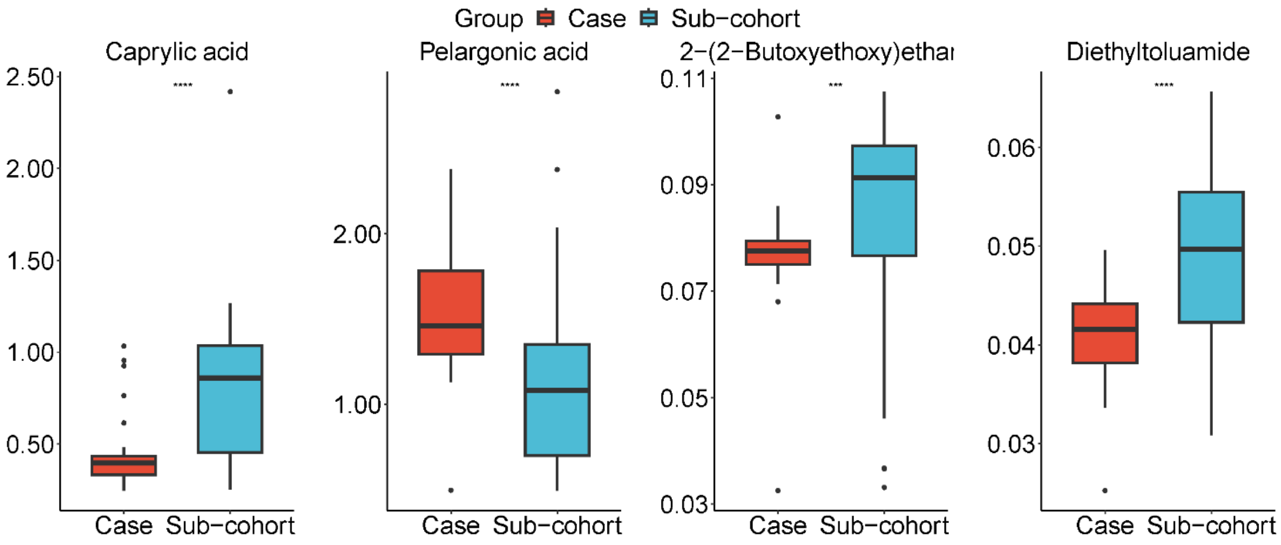


**Figure S5.** Boxplots showing levels of 4 differential metabolites in serum samples of GC case group and sub-cohort group.

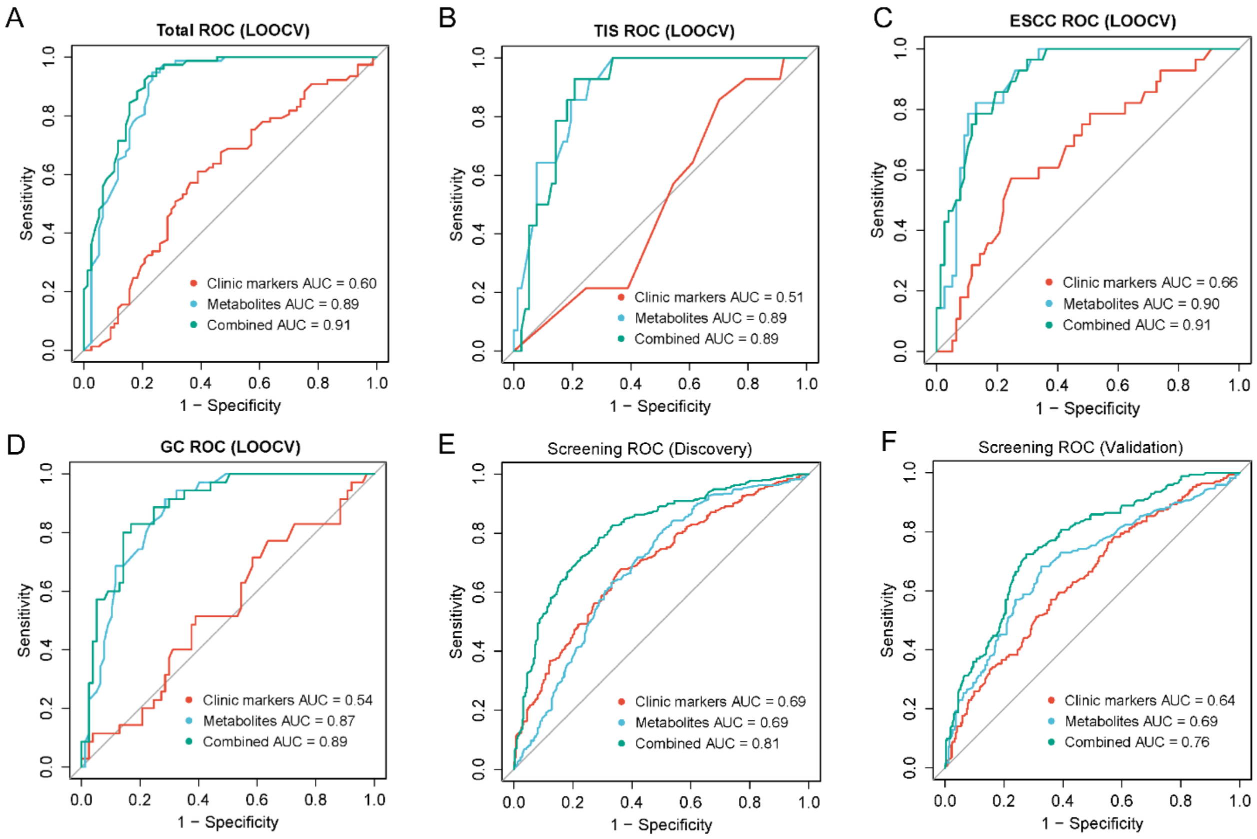


**Figure S6.** ROC curves of prediction models in the case-cohort study and ESCC screening study. (A) Random forest models to discriminate gastroesophageal cancer; (B) Random forest models to discriminate tumor in situ; (C) Random forest models to discriminate esophageal cancer; (D) Random forest models to discriminate gastric cancer; (E) ROC curves in discovery dataset; (F) ROC curves in validation dataset.
